# TRAF6 Inhibitors from Marine Compound Library: Pharmacophore, Virtual Screening, Fragment Replacement, ADMET, and Molecular Dynamics

**DOI:** 10.3390/md22060260

**Published:** 2024-06-05

**Authors:** Xuexuan Wu, Saiyi Zhong, Nan Zhou, Lianxiang Luo

**Affiliations:** 1 The First Clinical College, Guangdong Medical University, Zhanjiang 524023, China; wuxuexuan@gumu.edu.cn (X.W.); zaonan@gdmu.edu.cn (N.Z.); 2 Guangdong Provincial Key Laboratory of Aquatic Product Processing and Safety, Guangdong Province Engineering Laboratory for Marine Biological Products, Guangdong Provincial Engineering Technology Research Center of Seafood, College of Food Science and Technology, Guangdong Ocean University, Zhanjiang 524088, China; zhongsy@gdou.edu.cn; 3 The Marine Biomedical Research Institute of Guangdong Zhanjiang, School of Ocean and Tropical Medicine, Guangdong Medical University, Zhanjiang 524023, China

**Keywords:** TRAF6, marine natural compound, pharmacophore, molecular docking, fragments replace virtual screening, molecular dynamics

## Abstract

TRAF6 is an E3 ubiquitin ligase that plays a crucial role in cell signaling. It is known that MMP is involved in tumor metastasis, and TRAF6 induces MMP-9 expression by binding to BSG. However, inhibiting TRAF6’s ubiquitinase activity without disrupting the RING domain is a challenge that requires further research. To address this, we conducted computer-based drug screening to identify potential TRAF6 inhibitors. Using a ligand–receptor complex pharmacophore based on the inhibitor EGCG, known for its anti-tumor properties, we screened 52,765 marine compounds. After the molecular docking of 405 molecules with TRAF6, six compounds were selected for further analysis. By replacing fragments of non-binding compounds and conducting second docking, we identified two promising molecules, CMNPD9212-16 and CMNPD12791-8, with strong binding activity and favorable pharmacological properties. ADME and toxicity predictions confirmed their potential as TRAF6 inhibitors. Molecular dynamics simulations showed that CMNPD12791-8 maintained a stable structure with the target protein, comparable to EGCG. Therefore, CMNPD12791-8 holds promise as a potential inhibitor of TRAF6 for inhibiting tumor growth and metastasis.

## 1. Introduction

**Figure 1:**
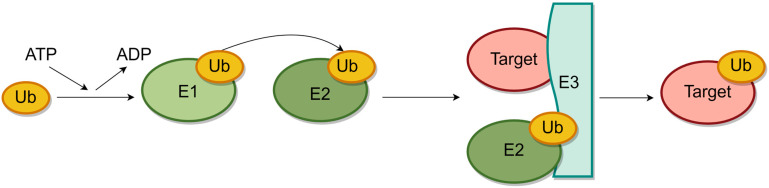
The process of ubiquitination. The first step is the ATP-dependent E1 activation of ubiquitin. The second step is to transfer the activated ubiquitin to the E2 enzyme to form the E2-Ub complex. Finally, E3 enzyme promotes ubiquitin transfer to the target protein. (By Figdraw2.0).

Ubiquitination plays a crucial role in preserving the stability of the majority of proteins within cells, regulating numerous eukaryotic pathway signals, and being linked to a range of pathophysiological cell disorders. This process is integral to cell cycle advancement, signal transduction, and membrane protein transportation, serving as a significant form of post-translational protein modification [1]. The enzymatic reaction of ubiquitination is carried out through the sequential action of the ubiquitin-activating enzyme E1, the ubiquitin-binding enzyme E2, and the ubiquitin–protein ligase E3 [2,3]. E1 initiates the activation of ubiquitin, followed by the transfer of the activated ubiquitin to E2 to create the E2-Ub complex. Subsequently, E3 serves as a mediator to facilitate the transfer of ubiquitin from E2 to the target protein, ultimately leading to ubiquitination [4,5]. Figure 1 illustrates this ubiquitin cascade process. Over the past few years, the ubiquitin–protein ligase E3 has garnered significant interest for its role in the ubiquitination cascade and its potential as a targeted inhibitor of ubiquitination [6].

Tumor necrosis factor receptor-associated factor 6 (TRAF6) belongs to the TRAF family, specifically the tumor necrosis factor receptor-associated factor. TRAF6 influences cancer signaling pathways, particularly the NF-κB pathway and MAPK pathway, thereby controlling tumor cell growth, viability, and invasion [7,8]. TRAF6 consists of an N-terminal RING domain, multiple zinc finger structures, and a C-terminal TRAF domain [9]. The C-terminal domain is where TRAF6 binds to its target protein, while the N-terminal domain displays strong ubiquitin-binding enzyme activity [10,11,12]. TRAF6, possessing ubiquitin ligase activity, plays a crucial role in facilitating LYS63 (K63)-linked polyubiquitinated chains. Working in conjunction with the E2 Ubc13/Uev1A complex, TRAF6 is responsible for triggering IKK activation, ultimately resulting in the activation of the NF-κB signal transduction pathway [11,13]. TRAF6 has been found to interact with Ubc13 at specific sites, including GLN54, ILE72, ASP57, and LUE74. When mutations occur at these sites, Ubc13’s ability to bind to TRAF6 is compromised [11,14]. Research has previously demonstrated TRAF6’s upregulation in various tumors and disease conditions, such as autoimmune diseases, liver cancer, and melanoma [15,16,17]. TRAF6’s association with tumors significantly impacts their development and prognosis, resulting in unfavorable outcomes [8]. The suppression of TRAF6 expression leads to a notable decrease in the malignant characteristics both in vitro and in vivo, underscoring the pivotal role of TRAF6 in tumor metastasis. This suggests that targeting TRAF6 could be an effective strategy for inhibiting tumor growth [17,18]. Currently, the significant failure rate of targeted inhibitors against TRAF6 has raised concerns about TRAF6-targeted inhibitors. This experiment aims to block the ubiquitinase activity of TRAF6 without destroying the N-terminal RING structure.

In recent years, the exploration of marine compounds has emerged as a new area of research, garnering increasing interest from scientists due to the limitations of land resources. Research has demonstrated that compounds derived from marine sources possess a range of beneficial effects, including anti-tumor, antibacterial, anti-inflammatory, and analgesic properties [19,20,21]. In comparison to terrestrial plants and non-marine microorganisms, marine organisms are regarded as a more recent source of bioactive products [22]. Marine organisms have undergone the longest evolution in a harsh living environment, which gives marine compounds unique chemical structures, amazing diversity, and high medicinal value. This means that marine compounds have more space to explore than terrestrial organisms [23]. Therefore, marine compounds have attracted more and more attention from scientists. Recent studies have shown that 170 marine compounds and their synthetic analogues have strong anti-cancer biological activity [24,25]. In our previous study, we successfully screened the target PD-L1, CDK4/6, and USP7-targeted inhibitors from the marine active compound library for the treatment and prevention of tumors [26,27,28,29]. Based on the broad potential of the marine compound library and our previous experience, it is possible to use marine natural compounds to screen potential inhibitors of TRAF6. In this research, we gathered information from three databases related to marine natural products: the Marine Natural Products Database (MNPD), Seaweed Metabolite Database (SWMD), and Comprehensive Marine Natural Products Database (CMNPD) [30,31,32]. Our goal is to leverage the rich resources of the ocean to identify novel TRAF6-targeted inhibitors.

**Figure 2:**
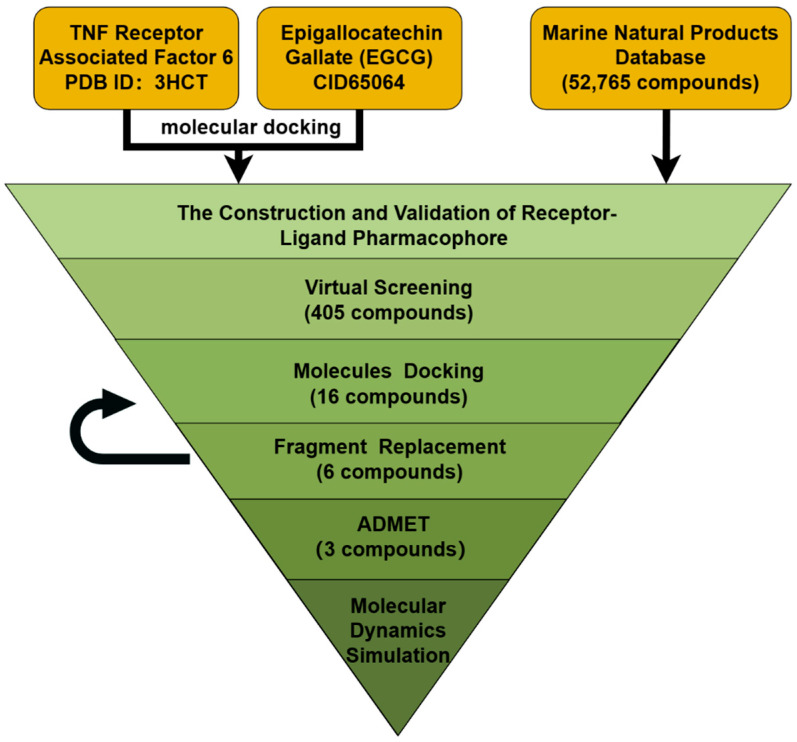
This work uses the workflow of computer virtual screening. It consists of pharmacophore modeling, virtual screening, molecular docking, fragment replacement, ADME prediction, toxicity prediction, and MD Simulation. (By Figdraw2.0).

In this study, our goal was to identify inhibitors that target TRAF6. We began by gathering information on the known molecule inhibitor of TRAF6 EGCG and conducted docking simulations based on the potential action sites GLN54 and ILE72. This allowed us to create a pharmacophore model for screening marine compound libraries. We analyzed the results of the docking simulations and selected promising compounds based on their scores and interactions. Next, we selected compounds that outperformed the positive controls for fragment replacement and compared the compounds before and after structural optimization through docking simulations and binding-free energy calculations. Subsequently, we conducted ADME and toxicity predictions, followed by molecular dynamics (MD) simulations, to identify potential TRAF6 inhibitors. The workflow of our study is illustrated in Figure 2.

## 2. Results

### 2.1. Molecular Docking of Known Inhibitors and TRAF6

**Figure 3:**
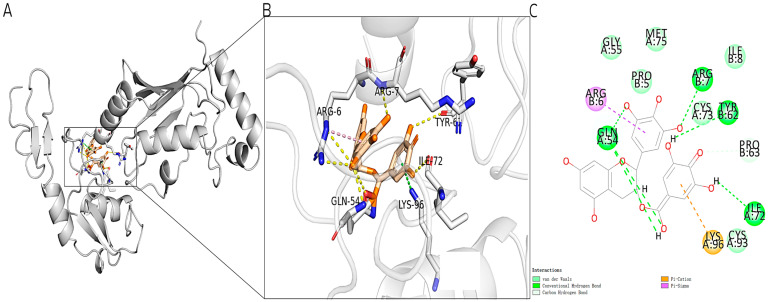
Molecular docking of known inhibitors EGCG and TRAF6. (**A**) The three-dimensional binding pattern of EGCG and TRAF6 molecular docking, α-helix for cylindrical drawing, β-helix for arrow drawing; (**B**) local amplification of EGCG and TRAF6 molecular docking; (**C**) the two-dimensional binding mode diagram of EGCG and TRAF6 molecular docking.

We conducted molecular docking using Discovery Studio 2019 Client to analyze the interaction between epigallocatechin-3-gallate (EGCG) and TRAF6. The binding sites GLN54 and ILE72 were identified as key sites for binding and were set as active binding sites. Through the docking process, we generated over 500 conformations and selected the ones that showed strong interactions with the key binding sites. The results indicated that EGCG binds to TRAF6 at key sites GLN54, LYS96, and ILE72 through various interactions, including hydrogen bonding, Π cation interaction, and Π-σ bonding (Figure 3). The docking score obtained was 98.886. Based on this successful docking complex, we plan to develop a ligand–receptor complex pharmacophore model.

### 2.2. Establishment and Validation of Ligand–Receptor Complex Pharmacophore

**Figure 4:**
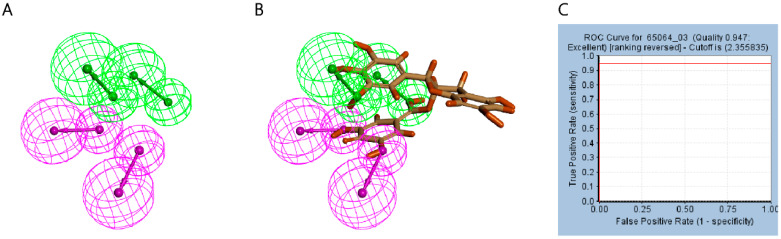
Establishment and validation of pharmacophore of ligand–receptor complex. (**A**) Pharmacophore_3, green represents hydrophobic feature and pink represents hydrogen bond donor feature; (**B**) EGCG and Pharmacophore _3’s mapping; (**C**) ROC curve of pharmacophore_3.

The pharmacophore is a representation of the spatial and electronic characteristics needed for ligands and macro-molecules to interact effectively. Following docking results, we utilized DS software to create six ligand–receptor complex pharmacophores. Among these, pharmacophore_3 was chosen for further screening after careful consideration (Figure 4A). Pharmacophore_3 includes a total of eight pharmacodynamic features, with four being hydrogen bond donors and four being hydrophobic. It is important to note that the chroman-5,7-diol structure of EGCG cannot be precisely matched with the pharmacophore when combined with pharmacophore_3, serving as a guide for the future virtual screening and fragment optimization of compounds (Figure 4B).

Validation is essential for selecting models that can accurately evaluate molecules. Before proceeding with further experiments, we confirmed the effectiveness of the pharmacophore in distinguishing between active and inactive molecules. The selected pharmacophore_3 achieved an AUC value of 0.947, indicating good recognition accuracy for future screening (Figure 4C). Additionally, the ROC curve generated from the verification process displays a somewhat flat second half, which could be a result of the limited dataset size. Despite this, the pharmacophore demonstrates a reliable ability to differentiate between active and inactive molecules, underscoring its effectiveness (Supplement Materials Table S1).

### 2.3. Virtual Screening

To initially identify molecules that interact with important binding sites, we utilized DS software to conduct a pharmacophore screening of numerous compounds. We combined three marine compound libraries, totaling 52,725 molecules, and subjected them to screening using pharmacophore_3. Molecules that matched the pharmacophore characteristics were kept, while those that did not were eliminated. By comparing the fit scores of the retained molecules with positive compounds, we identified 983 molecules with higher scores than the positive compounds. We then applied Lipinski’s rule of five and Veber’s rule for additional screening.

Compounds that adhere to Lipinski’s rule of five and Veber’s rules tend to have better pharmacokinetic properties and higher bioavailability in organisms. In a study screening 983 cyclic compounds, only one rule violation was allowed. Out of the 405 compounds that passed this screening, 30 exceeded the molecular weight limit, 24 had logP values outside the specified range, 47 had more than five hydrogen bond donors, and 30 had a polar surface area exceeding 140 Å^2^ (Supplementary Materials Table S2).

### 2.4. Molecular Docking

Molecular docking is a method used for designing based on the characteristics of receptors and the interactions between ligands and receptors to predict their binding modes. This method can determine the optimal mode of action for compounds and proteins, serving as a valuable reference for clinical trials. In this study, we employed the LibDock module of DS software to dock 405 molecules, resulting in the generation of 36,100 different conformations. The docking score of the known inhibitor EGCG was 98.886, and molecules with docking scores equal to or lower than this value were selected for further analysis. Each of the retained molecules was individually examined for their binding modes with TRAF6, and those lacking strong van der Waals forces with key binding sites such as GLN54, ILE72, and LYS96, as well as those exhibiting poor binding modes with TRAF6, were excluded from consideration. Only the best conformation of each molecule was retained for subsequent experiments, resulting in the selection of six molecules for further investigation, as detailed in Table 1.

### 2.5. Fragment Replacement

Fragment replacement is a strategy used to enhance the physicochemical and pharmacological properties of a drug by modifying its original structure. In this study, we focused on replacing fragments in the compound based on its binding mode with TRAF6. By utilizing a database of approximately three million fragments in DS software, we identified poorly-binding fragments and replaced them with rotatable ones. Through a systematic process, we replaced six fragments in the molecule, generating new compounds with over 100 fragment bindings each. The selection of replacement fragments was guided by considerations such as molecular weight, topological polar surface area, and lipid solubility to improve the binding affinity with TRAF6 (Table 2).

**Figure 5:**
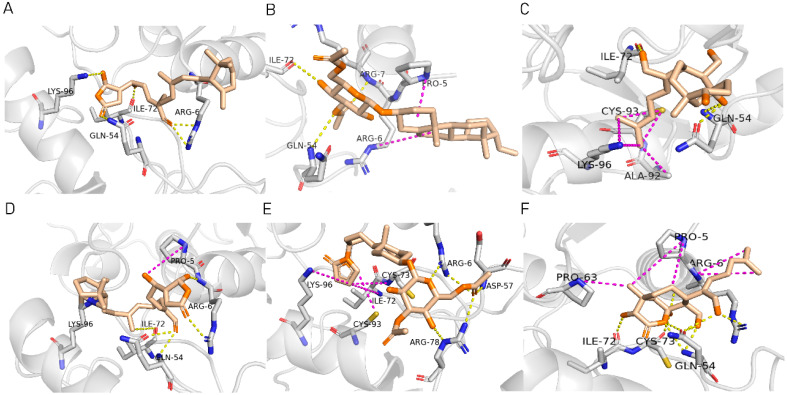
Molecule docking results before and after fragment replacement. (The yellow line represents hydrogen bond interaction. The powder line represents the hydrophobic interaction.) (**A**) Compound CMNPD12791 protein complex three-dimensional structure; (**B**) compound CMNPD26927 protein complex three-dimensional structure; (**C**) compound CMNP9212 protein complex three-dimensional structure; (**D**) structural optimization compound CMNPD12791-8 protein complex three-dimensional structure; (**E**) structured compound CMNPD26927-4 protein complex three-dimensional structure; (**F**) structural optimization compound protein complex CMNPD9212-16 three-dimensional structure.

To ensure the accuracy of our experiment and adhere to the principle of substitution, we conducted molecular re-docking to compare how different compounds bind to the key residues of TRAF6 before and after fragment replacement. Using high-throughput molecular docking software, we efficiently screened multiple molecules resulting from fragment replacement. After scoring and visually observing the results, we identified five groups of molecules with optimized structures. We studied the molecules before structural optimization and identified them according to their docking scores and binding residues for comparison with the molecules after structural optimization. Although CMNPD12791 interacts with key residues by hydrogen bonds, its 4,4-dimethyl-3-(3-methylbut-3-en-1-yl) cyclohex-1-enel does not interact with the protein (Figure 5A). CMNPD26927 is not well paired with the target residue and its 1-ethenyl-2-methylcyclopentane is exposed outside the active pocket of the protein (Figure 5B). CMNPD9212 mainly interacts with proteins in a hydrophobic interaction with fewer hydrogen bonds (Figure 5C). It is worth mentioning that CMNPD9212 has an alkanediol structure similar to the positive compound EGCG, and this structure has less interaction with TRAF6 on both EGCG and CMNPD9212. We focused on selecting molecules that closely resembled the original ones before structural optimization. Furthermore, we calculated the binding-free energy for both before and after fragment replacement, as well as for the positive compound. This energy calculation helps determine the potential affinity between the ligand and the target, with lower binding-free energy indicating a more stable complex (Table 3).

**Figure 6:**
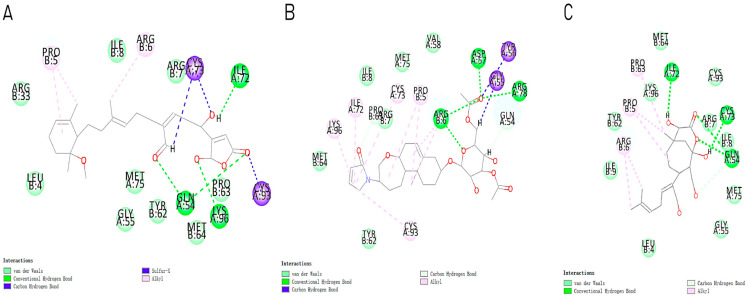
Molecular docking 2D image after fragment replacement. (**A**) Two-dimensional images of the interaction between compound CMNPD12791-8 and protein; (**B**) two-dimensional images of the interaction between compound CMNPD26927-4 and protein; (**C**) two-dimensional images of the interaction between compound CMNPD9212-16 and protein.

For the compounds CMNPD12791-8, the main interactions are mainly hydrogen bonds (including hydrogen bonds and carbon–hydrogen bonds). The residues GLN54, ILE72, LYS96, CYS73, and CYS93 are key residues that are bound to ligands by hydrogen bonds. The covalent binding of CMNPD12791-8 to LYS96, a key residue, is more stable than hydrogen bonding. At the same time, the residues PRO5, ARG6, and the oxygen atoms on the ligand form a hydrophobic interaction (Figure 6A). The 3D diagram of ligand protein docking shows that CMNPD12791-8 is basically completely embedded in the protein active pocket. This makes the protein interact with more residues by van der Waals force, and the ligand binds to the protein more firmly. (Figure 5D). It is important to note that the structurally optimized compound CMNPD26927-4 not only interact with GLN54, ARG78, GLN55, and TYR56 by hydrogen bonding, but also exhibit hydrogen bond interaction with ASP57, a critical site for TRAF6 binding. If ASP57 is mutated, the E2 ubiquitinase will lose its ability to bind to TRAF6 effectively (Figure 5E). In addition, the residues ILE72, LYS96, CYS73, CYS93, and the C atom of 1H-pyrrol-2 (5H)-one form hydrophobic interactions, and PRO3 and structures of oxoheptanes also form hydrophobic interactions, which facilitate further receptor–ligand binding (Figure 6B). When the cyclohexane-1,4-diol structure of CMNPD9212 was replaced by the (3S, 6R)-3,6-dihydroxytetrahydro-2H-pyran-2-one structure, the number of interactions with the protein greatly increased (Figure 6C). CMNPD9212-16 binds to GLN54, ILE72, and CYS73 by hydrogen bonding and forms hydrophobic interaction with PRO63, showing a more stable binding than the compounds before structural optimization. At the same time, CMNPD9212-16 is superior to CMNPD9212 in docking mode and scoring, which means that the results of our fragment substitution are reliable (Figure 5F). Following the replacement of fragments, all molecules exhibited enhanced performance in calculating docking scores and binding-free energy. Consequently, the optimized compounds CMNPD12791-8, CMNPD9212-16, and CMNPD26927-4 proved to be superior to the original ligands and positive compounds.

### 2.6. ADME Prediction

**Figure 7:**
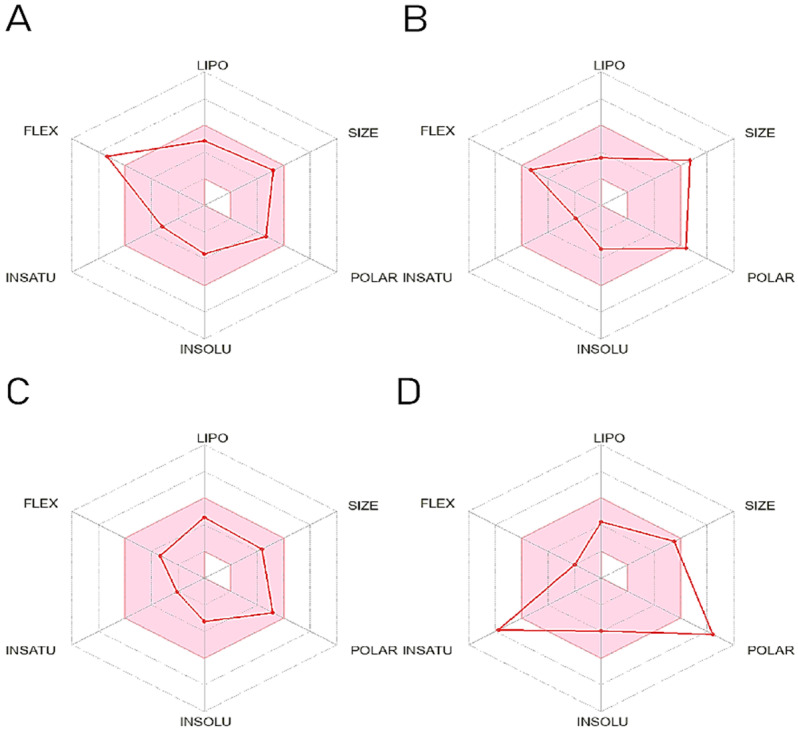
Hexagon diagram of ADME results of compounds. (**A**) ADME hexagon diagram of CMNPD12791-16; (**B**) ADME hexagon diagram of CMNPD29627-4; (**C**) ADME hexagon diagram of CMNPD9212-16; (**D**) ADME hexagon diagram of EGCG.

SwissADME is a free online tool that allows users to predict the pharmacokinetic properties of compounds by either drawing molecules or inputting SMILE numbers. Figure 7 displays the ADME hexagon distribution map of four molecules, where molecules with broken lines in the red area indicate better pharmacokinetic properties. Table 4 presents the gastrointestinal solubility, blood–brain barrier permeability, metabolizability, excretion, and drug-like properties of three compounds. Not all molecules are inhibitors of CYP2D6, indicating the good metabolic properties of the drug. Furthermore, all molecules showed improved gastrointestinal solubility compared to positive molecules. The compounds CMNPD12791-8, CMNPD921-16, and EGCG are substrates of P-glycoprotein, suggesting potential reduced availability in vivo. However, CMNPD12791-8 and CMNPD921-16 had higher bioavailability scores than CMNPD29627-4 and EGCG, likely due to their low water solubility. Druglikeness, which assesses similarity to known drugs, was predicted using SwissADME by evaluating compliance with various rules. CMNPD9212-16 scored highest in drug similarity, indicating good drug-like properties.

### 2.7. Toxicity Prediction

Before implementing computer drug design in clinical practice, it is crucial to predict the toxicity of selected compounds. To further assess the toxicity and adverse reactions of the compounds, we utilized the online tool ProTox 3.0 to predict the hepatotoxicity, neurotoxicity, mutagenicity, immunotoxicity, and carcinogenicity of three molecules. As shown in Table 5, CMNPD12791-8 and CMNPD9212-16 showed no neurotoxicity or carcinogenicity, unlike CMNPD29627-4. Overall, CMNPD12791-8 and CMNPD9212-16 exhibited favorable pharmacological properties and were selected for further molecular dynamics simulations.

### 2.8. Molecular Dynamics

**Figure 8:**
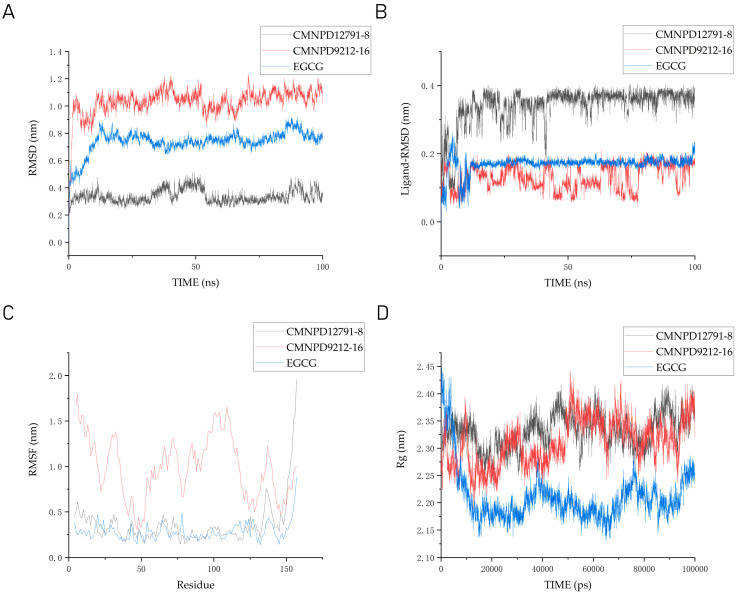
Analysis of molecular dynamics results. (**A**) RMSD analysis of the complexes formed by protein TRAF6 and three ligands respectively; (**B**) RMSD analysis of three ligands in protein TRAF6; (**C**) RMSF diagram of TRAF6 with compounds; (**D**) radius of gyration (Rg) graph for CMNPD9212-16, CMNPD12791-8, and EGCG complexes with respect to 50 ns of molecular dynamics.

The root mean square deviation (RMSD) measures the extent to which atoms deviate from their average position, indicating the level of motion of each atom. In Figure 8A, the RMSD results show that the TRAF6-CMNPD12791-8 complex reaches equilibrium at 0.5 ns and remains stable throughout the 100 ns simulation period. The average RMSD value is the smallest, at 0.3378 nm. The TRAF6-CMNPD9212-16 complex and the TRAF6-EGCG complex reached equilibrium at 8 ns, but the RMSD of the TRAF6-CMNPD9212-16 complex was larger than that of the TRAF6-EGCG complex, and the average value was higher. The average RMSD values of the RAF6-CMNPD9212-16 complex and the TRAF6-EGCG complex are 1.03203 nm and 0.73907 nm, respectively. Ligand RMSD analysis is shown in Figure 8B. The RMSD range of CMNPD12791-8 is between 0.2 and 0.4 nm, which is higher than that of CMNPD9212-16 and EGCG, which have an RMSD range of 0.1–0.3 nm. The average RMSD of CMNPD9212-16 is lower, at 0.13029 nm. In summary, the stability of the complexes formed by CMNPD12791-8, CMNPD9212-16, and protein is comparable to that of RAF6-TRAF6-EGCG complex.

The root mean square fluctuation (RMSF) of protein residues measures the extent of the movement of atoms within a protein conformation, indicating their degree of freedom. In proteins, the smaller RMSF means less flexibility in the molecular simulation process. As shown in Figure 8C, the RMSF value of TRAF6-CMNPD9212-16 complex is significantly higher than that of TRAF6-CMNPD12791-8 complex and TRAF6-EGCG complex. The RMSF trajectory of the TRAF6-CMNPD12791-8 complexes and the TRAF 6-EGCG complexes remained essentially consistent all along, with the range remaining between 0.2 and 1 nm.

The rigidity and compactness of complex structures are represented by the rotation radius (Rg) parameter. Therefore, in the presence of EGCG or inhibitors, the g_gyrate tool was used to monitor the compactness of the protein by the radius of rotation in GROMACS. In Figure 8D, the Rg range of TRAF6-CMNPD12791-8 complexes and TRAF6-CMNPD9212-16 complexes was consistent, ranging between 2.2 and 2.4 nm. The Rg of the TRAF 6-EGCG complexes ranged between 2.1 and 2.3 nm, but before 10 ns, the TRAF 6-EGCG complexes ranged between 2.3 and 2.45 nm. We can approximate that the Rg of the three is not much different.

**Figure 9:**
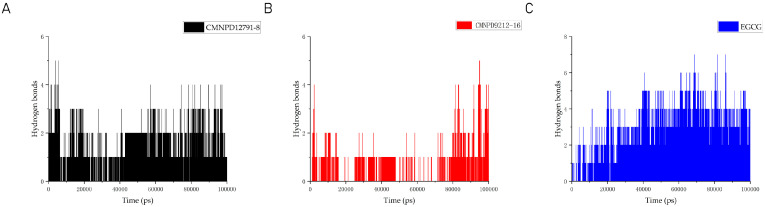
Hydrogen bonding between proteins and compounds. (**A**) CMNPD12791-8 with protein; (**B**) CMNPD9212-16 with protein; (**C**) EGCG with protein.

Hydrogen bonds are the strongest non-covalent interactions and play a crucial role in the stability of protein–ligand complexes. In our analysis of the number of hydrogen bonds in protein–ligand complexes and protein-positive compound complexes over a 100 ns molecular dynamics time span, we observed that CMNPD9212-16 has fewer hydrogen bonds compared to CMNPD12791-8 and the positive compound EGCG. As shown in Figure 9, the average number of hydrogen bonds of CMNPD12791-8 is 1.47576 and that of EGCG is 2.13413.

## 3. Discussion

TRAF6 is considered as a promising target for the development of anti-tumor drugs. Its presence is often associated with a poor prognosis in tumors. The current E3s inhibitors are mainly developed for HECT E3s. Compared with the RING structure, the HECT structure has potential catalytic activity [33,34]. However, some inhibitors of the RING structure are still in the preclinical stage (MDM2, IAP), and the success rate is limited [35,36]. This may be because they lack specificity or are limited by dose toxicity. Some TRAF6 receptor signal transduction inhibitors have been reported, including IRAK4 activity, Ubc13-Uev1a interaction, MYD88 dimerization, and Ubc13, but there are still limitations for targeting TRAF6’E3 ligase activity. The published molecule inhibitors targeting the E3 ligase C25-140 and resveratrol showed good effects on cells, but they still have problems such as limited bioavailability, poor stability, and large side effects, which pose challenges to clinical demand applications [37,38]. Hence, there is an urgent need to address these limitations and improve drug quality. Fortunately, computer technology has been developed to use CADD to screen molecules from large compound libraries to block TRAF6 without destroying the structure, making it a potential cancer treatment option.

In recent years, there has been a shift towards exploring marine natural products for drug development due to challenges of land-based-drug research. Many targeted drugs are now being derived from marine organisms, with compounds showing promise in areas such as antibacterial, anti-tumor, and anti-aging properties. The vast ocean breeds the beginning of life, and marine natural products have a rich diversity that is different from terrestrial products. Recently, many drugs extracted from marine natural products have been developed [28]. The process of selecting marine complex libraries leads to key aspects of new research.

In this research, we focused on the positive compound EGCG, which is a new inhibitor of E3 ligase targeting the N-terminal RING domain to potentially prevent and treat melanoma, lung cancer, and other tumors by targeting TRAF6. Previous studies have demonstrated that EGCG directly binds to TRAF6, inhibiting the binding of E3 to Ubc13 and consequently reducing the E3 ligase activity of TRAF6, leading to a weakening of tumor malignancy. In this study, we used EGCG and TRAF6 for docking, constructed the pharmacophore, and selected the 03 pharmacophore with a score of 0.947. We further validated the pharmacophore, and the area under the ROC curve indicated its ability to effectively discriminate. Using ChEMBL, we gathered 19 TRAF6 inhibitors to validate the pharmacophore_3 model. The AUC results showed that the pharmacophore had a strong discriminatory power. The second half of the ROC curve is flat; we believe that this may be the problem of too few data sets [39]. Nevertheless, because the pharmacophore can still distinguish between active and inactive molecules, we still believe that this pharmacophore is reliable [40,41]. From the mapping of pharmacophore and EGCG, we found that the chroman-5,7-diol structure of EGCG is difficult to interact with TRAF6 in non-covalent ways such as hydrogen bonding and hydrophobic interaction, which is not conducive to the binding of compounds to TRAF6. We used this pharmacophore to virtually screen the marine compound library, and the molecules that met the pharmacophore characteristics were retained. A total of 405 kinds of molecular docking were retrieved for further screening.

Quantifying the interaction between compounds and proteins is very important in computer-aided drug design. In a recent study, a marine natural compound library was utilized for virtual screening and molecular docking to identify potential inhibitors of TRAF6. Six molecules were identified, and through further evaluation and fragment replacement, three structurally optimized compounds—CMNPD12791-8, CMNPD9212-16, and CMNPD26927-4—showed improved docking scores and binding interactions with TRAF6. Among them, the structurally optimized compound 26927-4 and more key residues are effective, but they have poor drug-forming metabolisms and high toxicity. Compound CMNPD12791-8 optimized the 4,4-dimethyl-3-(3-methylbut-3-en-1-yl) cyclohex-1-ene structure at the tail end, thus forming a more stable direct binding with LYS96. We replaced the alkanediol structure similar to the positive compound CMNPD9212-16, which did not have a good effect on the protein. The subsequent molecular docking results showed that the new compound CMNPD9212-16 and TRAF6 were more closely combined after fragment replacement and achieved better docking scores and binding-free energy scores. Therefore, we believe that changing the alkanediol structure of the compound may improve the docking effect. All of the three structurally optimized compounds bind to the key residues GLN54 and ILE72 by hydrogen bonding or hydrophobic interaction. In addition, we found that the residues CYS73 and CYS93, which were not mentioned before, also play an important role in the interaction of ligand–receptor complexes. The optimized compounds also demonstrated better binding-free energy, indicating their ability to form stable complexes with the protein pocket. Overall, these findings support the rationality of fragment replacement in enhancing the binding affinity and stability of potential drug candidates derived from marine natural products. Molecular dynamics confirmed the static docking results. Despite fewer hydrogen bond interactions, CMNPD12791-8 showed promising binding properties, with CMNPD12791-8 emerging as a potentially superior candidate.

However, although we have predicted that the compounds CMNPD12791-8 have good pharmacokinetic properties and low carcinogenicity, this study still has some limitations, and further experimental data are needed to verify this inhibitory activity. In addition, further research is needed to verify free energy, which will be carried out in the future. In summary, we screened and optimized the potential targeted inhibitor CMNPD12791-8 of TRAF6 with excellent target binding potential from a rich marine compound library, which can be used for further clinical research and experimental reference for the development of TRAF6-targeted inhibitors.

## 4. Materials and Methods

### 4.1. Preparation of the Compound Data Set

In order to collect the reported biologically active TRAF6-targeted inhibitors, we collected 20 inhibitors from the publicly accessible ChEMBL website. In order to ensure the integrity of the compound data and to process the subsequent experimental steps more conveniently, we deleted the molecules with incomplete information and finally obtained 19 compounds. In this study, EGCG (CID65064) was used as a positive control because it can inhibit the phosphorylation of i-κba, block the nuclear translocation of p65 and p50, lead to the inactivation of the NF-κB pathway, and significantly inhibit the activity of TRAF6 E3 ubiquitin ligase, thereby inhibiting the growth and migration of tumor cells. For all compounds, we choose the CHARMn force field and use the “Prepare Ligand” module in the DS software to remove the salt and generate tautomers, set the standard charge on the common functional groups, and generate the corresponding energy-minimized 3D conformation.

### 4.2. Protein Preparation and Molecular Docking

We downloaded TRAF6 (PDB ID: 3HCT) from PDB. In order to give the protein a more accurate structure for subsequent experiments, we used the “Clean Protein” module of the DS software to remove the protein conformation, supplement the incomplete amino acid residues, and hydrogenate the protein, while manually removing the water molecules contained in the protein. We will use the prepared small molecular structure and protein to further screen TRAF6 inhibitors. Molecular docking is performed using the “Flexible Docking” module in the DS software. We selected the key residues GLN54 and ILE72 of TRAF6, which are now reported, and set them as flexible docking sites to generate a docking sphere with a radius of 11.3. Other parameters remain default. This means that the molecular conformation of the protein side chain and EGCG near these two residues is allowed to change to more accurately align the ligand to the protein residue. We calculated the root mean square deviation of the main ligand and its generated conformation, and then combined the docking scores of different conformations and the docking of key sites GLN54 and ILE72 to select the best docking results.

### 4.3. Establishment and Validation of Ligand–Receptor Complex Pharmacophore

Following recent developments, the establishment of l receptor–ligand pharmacophores has become a commonly used computer drug screening method that can accurately select the key binding sites of the receptor. The pharmacophore based on the ligand–receptor complex is a pharmacophore model generated according to the binding sites and binding modes of ligands and receptors, with high selectivity and high screening efficiency. We calculated the types of action modes of ligands and receptors and selected hydrogen bond donor binding, hydrophobic binding, and hydrogen bond receptor binding as the primary choices for generating pharmacophores. We set the energy threshold to 20 Å, and only six pharmacophores can be generated for reference. In order to verify that the pharmacophore can well distinguish between active and inactive molecules, we used the 19 inhibitor molecules found as active molecules and the open-source free website DUD-E to generate 18 decoy molecules as inactive molecules for testing. The receiver operating characteristic (ROC) curve, which shows the relationship between specificity and sensitivity, was used for this validation. The sensitivity is represented on the vertical axis, with higher values indicating better pharmacophore recognition. The horizontal axis represents specificity, with lower values indicating a lower false alarm rate. We used these 37 molecules to verify the reliability of the pharmacophore, plotted the ROC curve, and selected the best-performing pharmacophore_3.

### 4.4. Virtual Screening

Preliminary screening based on the ligand–receptor complex pharmacophores can quickly and accurately screen out bioactive molecules with specific targets but different structures in a large number of compound libraries. We import the prepared marine compound library into the DS software and use the “Prepare Ligand” module to generate multi-conformations, so that each molecule has a maximum of 255 conformations for subsequent screening. We set the energy threshold of pharmacophore matching to 20 Å. In addition to analyzing the screening fit score, we also visually examined the matching patterns of each molecule and pharmacophore and screened better compounds than positive compounds. In order to screen the compounds more accurately, we introduced Lipinski’s rule of five and Veber’s rule. These two rules are guidelines for determining the druggability of compounds in modern drug discovery and are often used in the virtual screening of drug candidates to enhance efficiency and reduce costs. These two rules stipulate that the molecular weight is not more than 500 Da, the fat water partition coefficient is less than 5, the number of hydrogen bond acceptors is not more than 10, the number of hydrogen bond donors is not more than five, the number of rotatable bonds is not more than 10, and the topological polar surface area is not more than 140 Å^2^. We stipulate that each molecule can only violate one of the rules, and molecules that meet the criteria are retained for more accurate screening.

### 4.5. Molecular Docking

In order to further screen TRAF6 inhibitors, high-throughput docking screening of candidate compounds was performed based on TRAF6 (PDB ID:3HCT). We import pre-prepared TRAF6 and molecules into the DS software for docking. Before starting the docking, we focus on the key sites GLN54, ILE72, and LYS96 to generate a sphere. We set the center coordinates of the docking site to (−21.533, 19.6752, 20.3813). We determined the docking radius to be 12 and defined this sphere as the specified active site. In order to make the docking results more accurate, we set the docking preference to high quality, set the docking tolerance to 0.25, and set the space hot spot number to 100. In addition, the ligand conformation generation method was designated as the best operation to better observe the various docking postures of small molecules and TRAF6 by comparing the docking scores of the positive compounds and manually viewing the binding mode of each ligand and TRAF6. The selected molecules were put into the next analysis and optimization, and ChemDraw22.0.0 was used to predict the structural information of the six selected molecules.

### 4.6. Structural Optimization Based on Replacement of Molecular Fragments

#### 4.6.1. Fragment Replacement

Fragment replacement is a common strategy for the computer design of drug structure optimization. The shape and number of substitution fragments in fragment-based design can greatly affect the physicochemical properties, druggability, and biological activity of molecules.

In order to make molecules better connected to TRAF6 and to improve the success rate of candidate molecules, we use the “Fragment Replacement” module on the DS software to further optimize molecules. For the purpose of retaining the skeleton that binds well to TRAF6 and to optimize the fragment that binds poorly, we manually select the fragment that binds poorly to TRAF6 for each molecule to replace the fragment. Firstly, we study the two-dimensional display of the combination of docking presented by the DS software. Secondly, according to the ligand interaction determined by the DS software, we manually select the areas with large exposure area, small binding force, and few binding sites. As shown in Table 2, blue represents the target replacement fragment. The DS software has its own fragment database that included about three million fragments. This experiment uses DS’s own database.

#### 4.6.2. Molecular Docking and Binding-Free Energy Calculation after Fragment Replacement

We use TRAF6 to perform high-throughput screening of newly generated molecules to better determine the effect of binding to the original pocket and efficiently find the target molecule from the thousands of generated molecules. In order to compare with the original molecule more intuitively, we define the same binding site as the original docking and select the same parameters for screening. The binding-free energy is used to calculate the docking energy of the ligand and the protein. We chose the DS software to calculate the binding-free energy based on the master equation. This method assumes that binding-free energy comes from the contribution of non-energy terms and that there is no cross-interaction between the energy terms. The total binding-free energy is obtained by calculating and adding these energy terms. Before calculating the binding energy, we define the protein force field as CHARMn and minimize the ligand energy. We observed the residues that interact with the ligand and defined it as flexible, and other parameters remain default.

The binding-free energy obtained by the DS software can be obtained by the following equation: Energy Binding = Energy Complex − Energy Ligand − Energy Receptor.

### 4.7. ADME Prediction

Predicting the ADMET (absorption, distribution, metabolism, and excretion) of com pounds is very important in drug design. In order to explore the druggability of the newly-generated drug molecules, we imported the drug molecules into the free online website SwissADME (URL: SwissADME). The website provides parameters such as lipophilicity, water-soluble LogS, drug similarity rules, and medicinal chemical properties. Through the SMILE number of molecules, the website can detect not only the pharmacokinetic properties of molecules but also the chemical and drug-like properties of drugs. This is helpful to save the material and time costs of our subsequent experiments and speed up the research of TRAF6 inhibitors. A good gastrointestinal solubility score means that the drug can be taken orally and absorbed into the blood circulation through the intestinal wall. Central nervous system drugs need to have good blood–brain barrier permeability to allow molecules of drugs to reach the affected area. When the compound has a target in the central nervous system, blood–brain barrier permeability is crucial. P-glycoprotein is involved in xenobiotic outflow in the human body. The substrate of P-gp can be effluxed by cell surface proteins. Cytochrome P450 (CYP450) is involved in drug metabolism.

### 4.8. Toxicity Prediction

Predicting the toxicity of compounds is a common method for drug design that is helpful to predict the adverse reactions of drugs. We chose the online tool ProTox 3.0 to predict the toxicity of selected compounds, which provides a wide range of toxicity indicators such as hepatotoxicity, neurotoxicity, cytotoxicity, and immunotoxicity. In addition, it can also predict the toxicity of drugs to cell signaling pathways as well as the metabolic and blood–brain barrier permeability of drugs. We entered the SMILE number and manually adjusted some molecular structures to obtain the toxicity prediction results of the compounds.

### 4.9. Molecular Dynamics Simulations

In order to evaluate the binding stability of candidate molecules to protein TRAF6, molecular dynamics (MD) simulation was performed. Before running the MD, the simulation system was built using the GROMACS 2019.1 software package (provided by Mark Abraham et al.) [42,43]. The topological system of the protein was constructed using the AMBER99SB-ILDN force field. The Bio2Byte web server (accessed by https://www.bio2byte.be/, on 2 April 2023) is used to generate topology files for molecules. In the simulation system, the cubic box with a radius of 1.2 nm and the SPC216 water model are selected to define the periodic boundary conditions. At a simulated temperature of 300 K, the energy minimization of the system is carried out in 50,000 steps. After the energy is minimized, in order to maintain the pressure and temperature of the system, the position-constrained MD simulation is performed at 300 K (Kelvin) for a duration of 100 ps, and, finally, the MD simulation with a duration of 50 ns is performed. Through MD simulation, the trajectory coordinates are extracted, and the root mean square deviation (RMSD) and the root mean square fluctuation (RMSF) of the atomic position are analyzed. In addition, the gxo hbond analysis tool was used to extract the intermolecular H-bond interaction between TRAF6 and the ligands. At the same time, the radius of gyration of the protein ligand conjugates was calculated [44,45,46].

## 5. Conclusions

This study aimed to discover a new inhibitor that targets TRAF6 using computational methods. Three marine compound libraries were used to screen 52,765 molecules with a ligand–receptor complex pharmacophore. After conducting molecular docking on 405 selected compounds and comparing their scores with the positive compound EGCG, six potential molecules were identified based on the key inhibitory residues of TRAF6. The structures of these molecules were optimized by addressing fragments with suboptimal target effects. Molecular docking and binding-free energy calculations were then performed on the optimized compounds, comparing them to both EGCG and the original molecules. Three compounds were selected for further evaluation through ADME and toxicity predictions, followed by molecular dynamics simulation. The results showed that the protein-binding complexes of CMNPD12791-8 had comparable binding properties to those of EGCG, indicating promising potential for targeted tumor therapy.

## Figures and Tables

**Table 1:** The result of molecular docking.

Molecules	2D Structure	Libdock Score	Formula
CMNPD12791	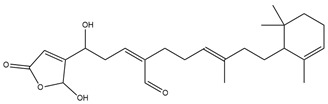	125.225	C_25_H_36_O_6_
CMNPD22985	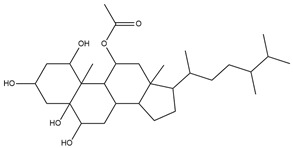	113.028	C_28_H_50_N_2_O_6_
CMNPD9212	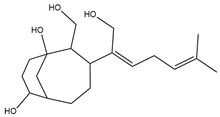	103.552	C_19_H_30_O_6_
CMNPD26927	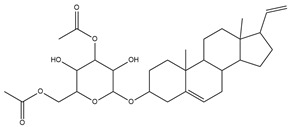	111.781	C_29_H_41_NO_10_
24831	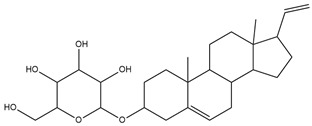	104.737	C_26_H_39_NO_7_
24987	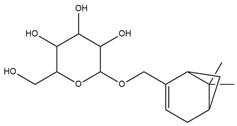	101.803	C_16_H_27_NO_6_

**Table 2:** The result of fragment replacement. (The replacement fragment is marked with blue.).

Molecules	Filter Criteria	Before Fragment Replacement	After Fragment Replacement
CMNPD12791-8	Weight < 500, Alogp [−6, 5], TPSA [40, 140]	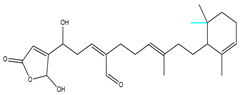	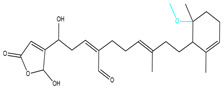
CMNPD22985-57	Weight < 600, AlogP [−6, 5], TPSA [40, 140]	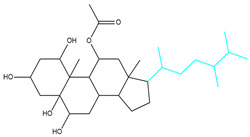	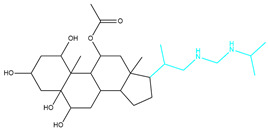
CMNPD26927-4	Weight < 500, AlogP [−6, 5], TPSA [40, 140]	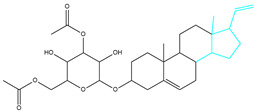	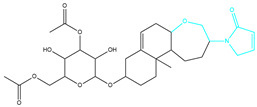
CMNPD9212-16	Weight < 500, AlogP [−6, 5], TPSA [40, 140]	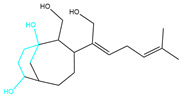	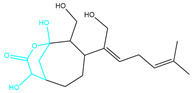
24831-1	Weight < 500, AlogP [−6, 5], TPSA [40, 140]	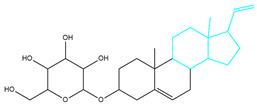	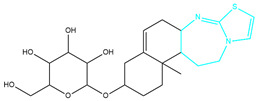
24831-4			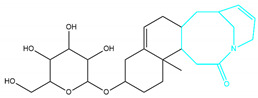

**Table 3:** Key role residues of compounds and proteins before and after fragment replacement.

Molecules	Hydrogen Bond	Hydrophobic Interaction	Pi-Cation	Score	Binding Energy (kcal/mol)
EGCG	ILE72, GLN54, CYS73	-	LYS96	98.882	−228.79
CMNPD12791	GLN54, LYS96, ILE72	-	-	125.225	−233.98
CMNPD12791-8	GLN54, ILE72	-	-	137.028	−334.67
CMNPD9212	GLN54, ILE72	CYS93, LYS96, ALA92	-	103.552	−195.85
CMNPD9212-16	GLN54, ILE72, CYS73	PRO63	-	109.351	−235.73
CMNPD26927	GLN54, ILE72	-	-	111.781	−240.92
CMNPD26927-4	GLN54, ASP57, ARG78	ILE72, LYS96, CYS73, CYS93	-	133.415	−338.23

**Table 4:** ADMET results for compound P217564 and the structural optimized compounds.

Molecules	GI Absorption	BBB Permeant	P-gp Substrate	CYP450 Inhibitor	Druglikeness	Bioavailability Score
CMNPD12791-8	High	No	Yes	No	4	0.55
CMNPD29627-4	High	No	No	No	1	0.17
CMNPD9212-16	High	No	Yes	No	5	0.55
EGCG	Low	No	Yes	No	1	0.17

GI absorption: gastrointestinal solubility; BBB permeant: blood–brain barrier permeability; P-gp substrate: is it a substrate of P glycoprotein; CYP450 inhibitor: is it an inhibitor of Cyt-450; Druglikeness: whether molecules comply with Lipinski’s rule of five, the Ghose rule, Verber’s rule, the Egan rule, and Muegge’s rule. We stipulate that compliance with one rule is one point, and compliance with more than two rules is considered to indicate better drug-like properties.

**Table 5:** Results of toxicity prediction.

Name	Hepatotoxicity	Neurotoxicity	Mutagenicity	Immunotoxicity	Carcinogenicity
CMNPD12791-8	No	No	No	No	No
CMNPD29627-4	No	Yes	No	No	Yes
CMNPD9212-16	No	No	No	No	No

## Data Availability

The data used to support the findings of this study are included within the article.

## Supplementary Materials

The following supporting information can be downloaded at: https://www.mdpi.com/article/10.3390/md22060260/s1, Table S1: The validation of active molecules and inactive molecules by the generated six pharmacophores. Table S2: 405 compounds after virtual screening.
